# An unexpected complication of nonoperative treatment for tibial posterior ­malleolus fracture: bony entrapment of tibialis posterior tendon – a case report

**DOI:** 10.1080/17453674.2019.1652972

**Published:** 2019-08-12

**Authors:** Thomas Amouyel, Baptiste Benazech, Marc Saab, Nadine Sturbois-Nachef, Carlos Maynou, Patrice Mertl

**Affiliations:** a Université de Lille Nord de France, Service d’orthopédie 1, Hôpital Roger Salengro, Centre Hospitalier Universitaire de Lille, France;; b Service orthopédie, Centre Hospitalo-Universitaire Amiens Picardie, 80480 Amiens, France

A 41-year-old patient was referred to our center because of right medial ankle pain increasing for 3 months. He had, 10 years ago, had a displaced lateral malleolus fracture with an associated non-displaced posterior malleolus fracture but without a medial malleolus fracture. A fibular osteosynthesis without medial or posterior exploration was done at another hospital. Postoperatively, the patient remained non-weightbearing for 6 weeks with a cast. The patient recovered completely and returned to work as a fireman 4 months after the initial injury. The fibular osteosynthesis material was removed 1 year after the surgery.

10 years later, when referred to our center, he had increasing medial ankle swelling and pain, preventing him from working as a fireman. Physical examination revealed a medial retromalleolar swelling with local tenderness, but no flat-foot deformity. Testing of the tibialis posterior tendon (TPT) was positive: heel-rise test and strength assessment were painful but with no loss of strength. Radiographs showed a healed lateral malleolus fracture in good alignment with a posterior tibial bony callus (Figure 1).

**Figure 1.**
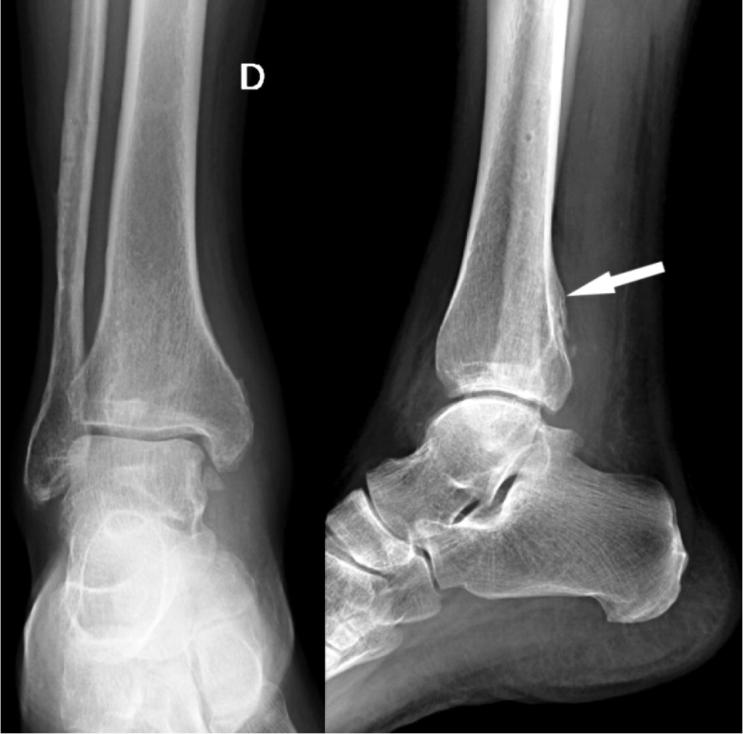
Posterior tibial bony callus (arrow) of the right ankle.

A CT scan showed a medial retromalleolar bone tunnel containing the TPT of 15 mm length (Figure 2); the fibular and the posterior malleolus fractures were healed. An MRI scan showed tenosynovitis of the TPT (Figure 3), and thickening of the anterior talo-fibular ligament.

**Figure 2.**
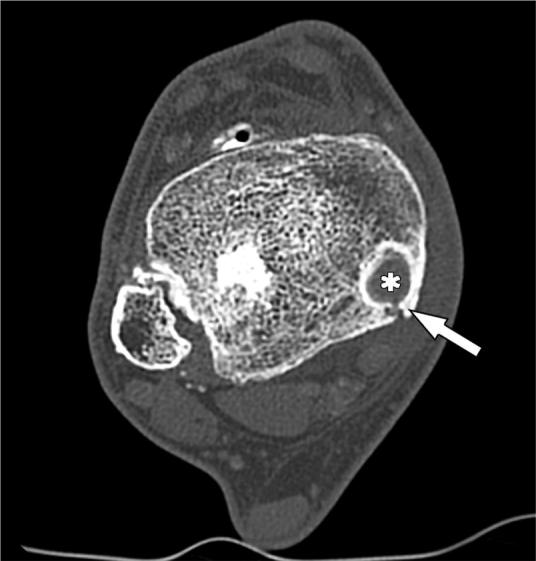
Ax CT scan, showing the tibialis posterior tendon (*) in a medial retromalleolar bone tunnel and the healed posterior malleolus fracture (red line).

**Figure 3.**
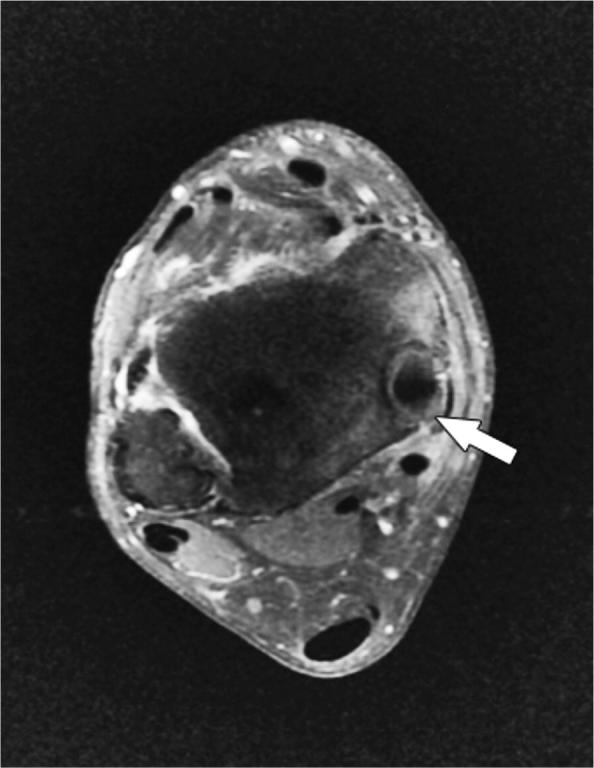
Tibialis posterior tendon tenosynovitis (arrow) on fat-saturation gadolinium injected T1-weighted axial MRI.

We performed an open resection of the postero-medial part of the tunnel to release the TPT (Figure 4). The postero-medial part of the bony tunnel was resected and the TPT was released, inspected, and debrided (Figure 5). The TPT moved freely in its groove with no tendency to luxation. Bone wax was pressed into cancellous bone to prevent recurrence of the bony tunnel.

**Figure 4.**
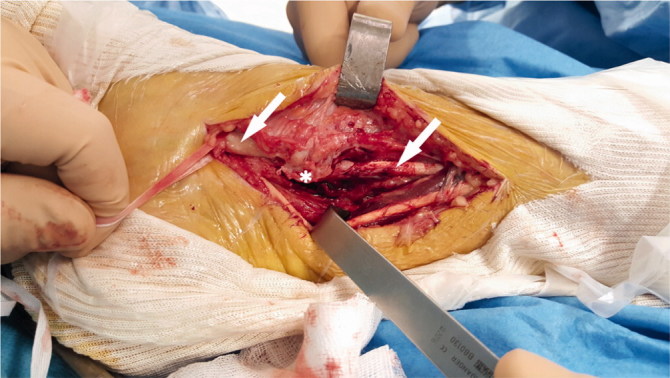
Tibialis posterior tendon identification above and below the medial malleolus (arrows), postero-medial part of the bony tunnel (*). (Right ankle, postero-medial approach, patient in supine position.)

**Figure 5.**
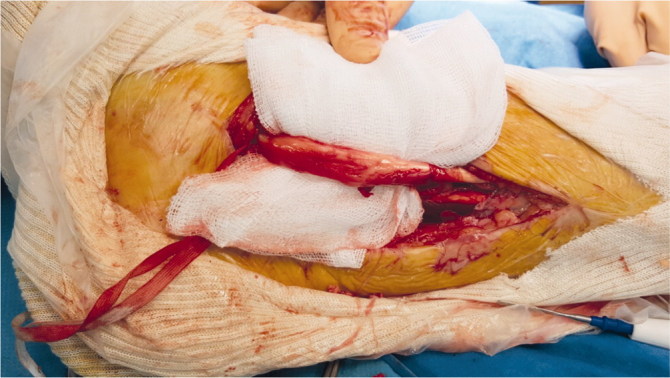
Tibialis posterior tendon debridement after resection of the retro-malleolar bone tunnel. (Right ankle, postero-medial approach, patient in supine position.)

The patient had a walking brace for 3 weeks and functional rehabilitation was started a few days after the surgery. At 6 weeks, the patient could walk with normal shoes and he was able to return to work after 3 months. At last follow-up (12 months), the patient had no pain and had returned to sport without physical limitation.

## Discussion

Tendon entrapment in bony callus is a rare complication of closed-reduction fracture management. Tendon is usually trapped directly in the fracture preventing its anatomical reduction, but it can also be engulfed in the growing osseous callus (Christodoulou et al. 2005, Erra et al. 2013).

While displaced medial and lateral malleolus fractures are often operated on, allowing the diagnosis of the tendon entrapment, posterior malleolus fractures are often neglected or fixed with anterior to posterior screws through a percutaneous approach (Solan and Sakellariou 2017). Internal fixation seems recommended for posterior malleolus fractures involving more than 25% of the articular surface to achieve anatomical reduction (Gardner et al. 2011, Mingo-Robinet et al. 2011). Surgery via a postero-lateral or postero-medial approach allows for anatomical reduction and direct control of tendon and soft tissue entrapment, and thus reduces the risk of malunion. Recent research articles showed good results in patients with posterior malleolus synthesis by screw or buttress plate, without increasing the complication rate due to the postero-lateral approach (Verhage et al. 2016, Bali et al. 2017, Gougoulias and Sakellariou 2017).

Structure entrapment is better known after upper limb fractures. Tendon entrapment has been reported rate in 1.3% of distal radius fractures involving particularly the extensor tendon and sometimes flexor tendon (Okazaki et al. 2009). Peripheral nerves can also be engulfed in fracture callus (Erra et al. 2013).

In our case, the TPT retromalleolar groove was closed by the posterior malleolus fracture’s bony callus, but with no symptoms for almost 10 years. It probably became painful due to a conflict within the inextensible groove, resulting in a painful tenosynovitis. We found 1 similar case in the literature but the entrapment was not circumferential and it concerned a medial malleolus fracture treated nonoperatively (Khamaisy et al. 2012). The treatment and the outcomes were similar in each case, both patients returning fully to their former activities.
